# Exploring the micromorphological characteristics of adult lower cervical vertebrae based on micro-computed tomography

**DOI:** 10.1038/s41598-023-39703-4

**Published:** 2023-07-31

**Authors:** Kun Li, Yang Yang, Peng Wang, Haoyu Song, Chunying Ma, Yansong Zhang, Xingye Dang, Jun Shi, Shaojie Zhang, Zhijun Li, Xing Wang

**Affiliations:** 1 School of Traditional Chinese Medicine, Beijing University of Chinese Medicine, Beijing, 100029 China; 2 Human Anatomy Teaching and Research Section, School of Basic Medicine, Inner Mongolia Medical University, Hohhot, 010059 Inner Mongolia China; 3 Graduate School, Inner Mongolia Medical University, Hohhot, 010059 Inner Mongolia China; 4 School of Clinical Medicine, Inner Mongolia Medical University, Hohhot, 010059 Inner Mongolia China; 5 Physiology Teaching and Research Section, School of Basic Medicine, Inner Mongolia Medical University, Hohhot, 010059 Inner Mongolia China; 6 Digital Medicine Center, School of Basic Medicine, Inner Mongolia Medical University, Hohhot, 010059 Inner Mongolia China

**Keywords:** Anatomy, Medical research

## Abstract

We will use micro-computed tomography to scan 31 sets of the adult lower cervical vertebrae (155 vertebrae) to observe the morphological characteristics and direction of trabeculae in the lower cervical vertebrae by outlining and reconstructing the regions of interest and to calculate the variation laws of the microstructure in the regions of interest to reveal their structural characteristics and weak areas. As a result, the images showed that the trabeculae in the lower cervical pedicle near the medial and lateral cortices were relatively dense, and their bone plates were lamellar. There were cavities between the superior and inferior articular processes where the ossification centers had not been absorbed after ossified. The lamellar trabeculae in the vertebral plates near the cortical bones were only 1–2 layers, extended and transformed into rod-shaped trabeculae in a radial shape toward the medullary space. The lamellar trabeculae of the vertebral plate extend over the spinous process near the cortical bone. The statistical results of the trabeculae's morphological parameters showed significant differences in bone volume fraction values among the four parts (P < 0.05). There were substantial differences in BS/BV, except for no differences between the pedicle and the vertebral plate (P < 0.05). There was a significant difference in trabecular pattern factor values between the articular process, the spinous process, and the vertebral plate (P < 0.05) and a significant difference between the pedicle, the spinous process, and the vertebral plate (P < 0.05). There were no significant differences in trabecular bone thickness and trabecular space values among the four parts (P < 0.05). The anatomical microstructural perspective confirms that the optimal choice is internal fixation via the pedicle. If using pedicle screws, the nail tract needs to be placed into the spinous process to increase its holding power and resistance to extraction.

## Introduction

As an essential structure to bear the head's weight, the lower cervical vertebra is the main spinal segment that causes the neck to protrude forward and form physiologic curvature. Cervical curvature supports the lifting of the head and increases the elasticity of the spine. It is of great significance in maintaining the stability of the body's gravity center and reducing concussions. Due to changes in people's work and lifestyle, the incidence of cervical spine disorders is increasing and is gradually becoming younger^1–3^. When the injury compresses the cervical cord or peripheral nerves, the clinical treatment is usually performed by internal fixation via screws to restore stability, including internal fixation via the pedicle, the articular process, or the vertebral plate^4–6^. Currently, most studies on lower cervical internal fixation have focused on its stability from the biomechanical point of view but less from the anatomical point of view of the vertebrae themselves^7–9^.

Conventional computed tomography can detect early injury to the lower cervical spine but cannot observe the microstructure because of its low resolution. Micro-computed tomography(Micro-CT), is a non-destructive, high-resolution imaging method developed recently, with resolution up to the micron level. Micro-CT has been widely used in osteology. Furthermore, scholars at home and abroad have applied it to analyze bone microstructure with osteoporosis and other diseases^10–13^. However, few studies have used Micro-CT to observe bone microarchitecture to guide clinical procedure selection.

In this study, we intend to take the perspective of the anatomical microstructure of vertebrae. After selecting the region of interest (ROI) of the lower cervical spine, we used Micro-CT to investigate their trabecular structural characteristics and alignment patterns. Then by observing their morphological characteristics to evaluate their overall three-dimensional structure and analyzing their trabecular parameters to accurately describe the bony characteristics of each part of the lower cervical spine. It will provide an anatomical basis for selecting clinical internal fixation procedures and a theoretical basis for further research on the biomechanical characteristics of the lower cervical spine and clinical and surgical treatment strategies.

## Methods

### Designates

The lower cervical pedicle, articular process, vertebral plate, and spinous process were selected as the ROI. Then using Micro-CT to observe the trabecular alignment and distribution patterns and to measure the bone structural parameters.

### Time and location

The experiment was completed in the Center for Digital Medicine of Inner Mongolia Medical University from March to June 2022.

### Human sample

The Human Anatomy Laboratory of Inner Mongolia Medical University provided the Chinese adult dried bone specimens used in this study. All met the experimental study requirements of the bone anthropology identification standard. Excluding bone specimens with bone destruction and defects, thirty-one sets of standard lower cervical skeletal specimens were selected, including 31 each of C3, C4, C5, C6, and C7, for a total of 155 vertebrae (310 pedicles, articular processes, and plates, and 155 spinous processes). Micro-CT scans to observe the microstructure and morphometric parameters of the trabeculae within the vertebral arches, articular processes, laminae and spinous processes. The study was approved by the Medical Ethics Committee of Inner Mongolia Medical University. The statement confirms that all methods were performed per relevant guidelines and regulations.

### Imaging

Micro-CT scan: Micro-CT based LBF model extraction and feature analysis of the lower cervical trabeculae Hiscan XM Micro CT (Suzhou Hesfid Information Technology Co., Ltd., Hiscan XM Micro CT) and its own analysis and measurement software (Hiscan Analyzer software) were analyzed. The scan parameters were: layer thickness 0.05 mm, layer spacing 0.05 mm, single exposure time 50 ms, bulb voltage 60 kV, current 134 µA, 2000 × 1600 matrix imaging, scan imaging field of view 10 cm × 8 cm, pixel size 0.05 × 0.05, and Dicom images were acquired and stored by Lenovo P320 workstation completed and stored, provided by Suzhou Hesfed Information Technology Co.

### Observations and measurements

After importing the data into the Inveon Research Workplace main interface, the ROIs were drawn manually in the horizontal 2D images, including pedicle, articular processes, lamina, and spinous processes. Moreover, all ROIs were drawn with different contours (rectangles, circles, ellipses, irregular polygons) based on the morphology of the structures presented in the processed images. We observed the morphological characteristics of the trabeculae in the coronal, transverse, and sagittal planes presented by the software and in the 3D reconstructed images, focusing on the description and analysis of the ROI's microscopic morphology. By using the self-contained program to calculate the parameters of the trabeculae in ROIs, the parameters of the calculated region of interest can be seen in Table 1.

**Table 1:** Description and definition of cancellous bone 3D results.

Variables	Definitions
Bone volume/total volume (BV/TV)	Ratio of bone trabecular volume to region of interest volume
Bone surface/bone volume (BS/BV, mm^−1^)	Ratio of bone surface area to bone volume in region of interest
Bone surface density (BS/TB, mm^–1^)	Ratio of bone surface area to total volume in region of interest
Trabecular thickness (Tb.Th, mm)	Average thickness of bone trabeculae measured directly using the 3D method
Trabecular number (Tb.N, mm^–1^)	Average number of trabeculae per unit length
Trabecular separation (Tb. Sp,mm)	Average distance between bone trabeculae measured directly using the 3D method
Degree of anisotropy (DA)	1 = isotropy, > 1 = non-isotropy; DA equal to the longest intercept length divided by the shortest intercept length
Trabecular bone pattern factor (Tb. Pf, mm^–1^)	The smaller the value of the parameter of bone trabecular connectivity, the better the connectivity of bone trabeculae

### Statistical analysis

The obtained data were inputted into the EXCEL (Microsoft Corp., Redmond, WA, USA) and analyzed by IBMSPSS21.0 statistical software (IBM Corp., Armonk, NY, USA). The measurement data were expressed as average ± standard deviation (x ± s), paired t-tests were used for comparisons between the right and left sides of the vertebral arch, articular eminence, and vertebral plate, and one-way ANOVA was used for comparisons between the C3–C7 groups for the vertebral arch, articular eminence, vertebral plate, and spinous process. The test level was established as a = 0.05, and the significant difference was considered at P < 0.05.

## Results

### Microstructure morphology

**Figure 1:**
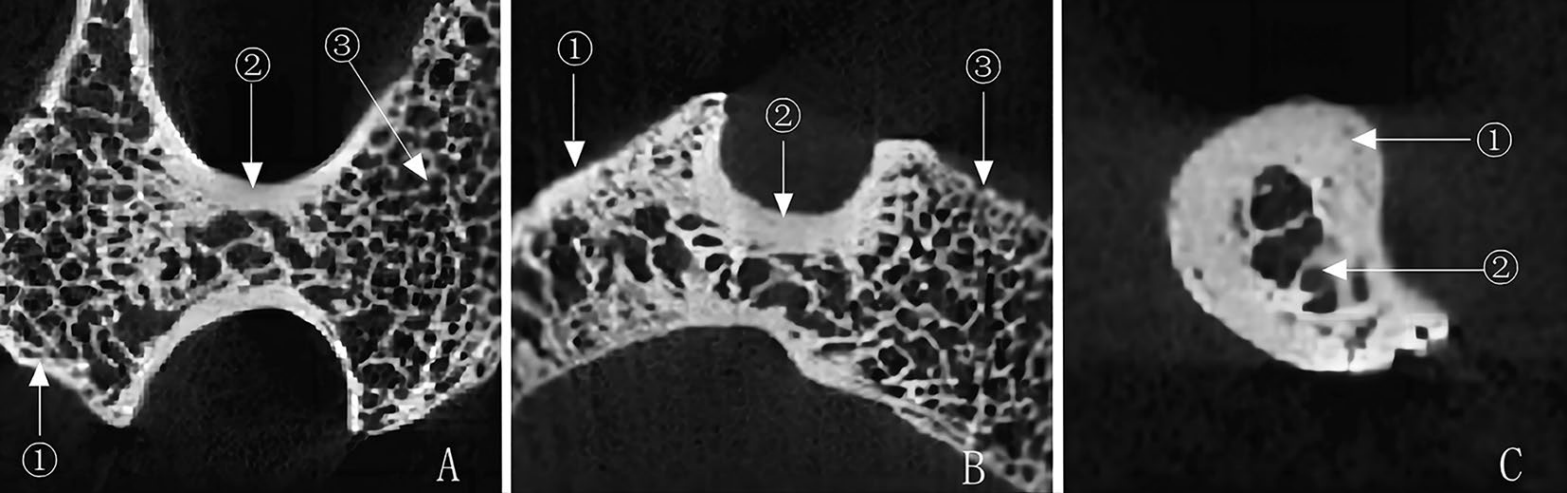
Trabecular arrangement characteristics within the pedicle (**A** Cross-section: ①articular process ② pedicle ③ vertebral body. **B** Sagittal plane: ① articular process ② pedicle ③ vertebral body; **C** Coronal section: ① cortical bone ② trabecular).

The images showed that the trabeculae near the medial and lateral cortex of the pedicle were lamellar, and the structure was relatively dense. The trabeculae in the medial cortical bone were significantly more than in the lateral cortical bone. The lamellar trabeculae extended to the anterior pyramidal part and the posterior lamina. Then it formed a solid reticular structure with oblique rod-shaped trabeculae in the medullary space. Within the cavum medullare, sparse rod-shaped trabecular distribution and complex netlike cellular trabecular structure (see Fig. 1).

**Figure 2:**
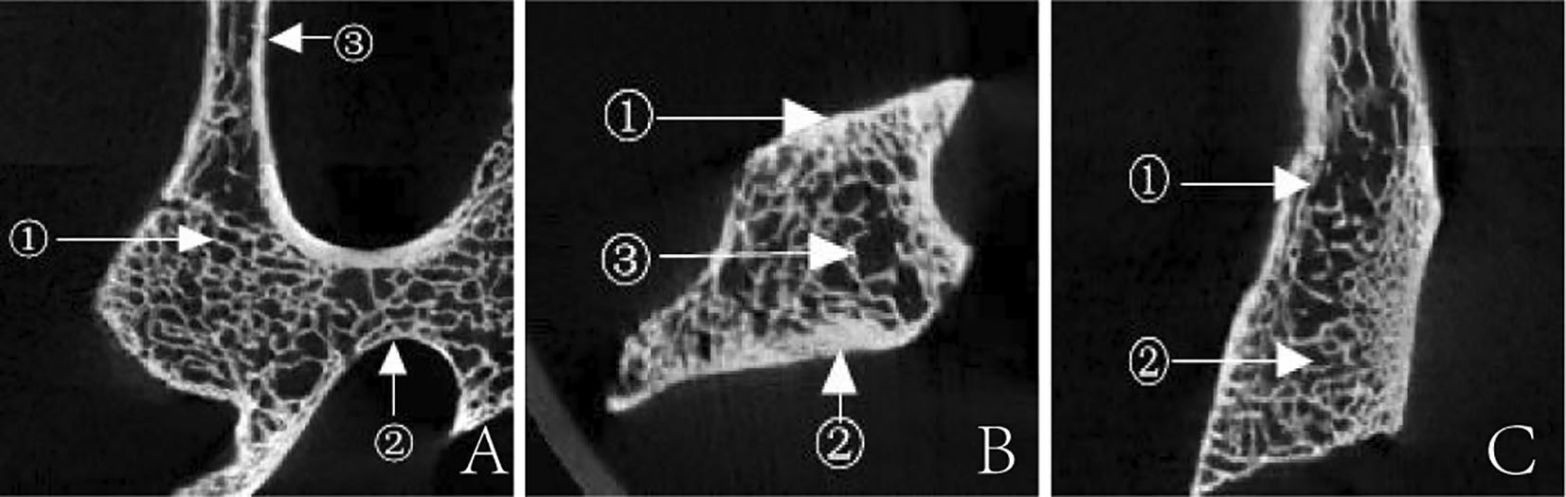
Trabecular arrangement characteristics within the articular process (**A** Cross-section: ① articular process ②pedicle. **B** Sagittal plane: ① superior articular process ② inferior articular process ③ trabecular cavity).

The unclosed ossification center between the superior and inferior articular process forms a trabecular cavity. In the cavity, its structure differs from the typical trabeculae. The cellular trabeculae of the articular process were also complex and reticular. The bone plate of the trabecular, which is close to the cortical bone, is lamellar and compact. It extends and transforms into a reticular structure, then a rod-shaped trabecula. The rod-shaped trabeculae converge with only 1–2 layers of lamellar trabeculae around the trabecular cavities (see Fig. 2).

**Figure 3:**
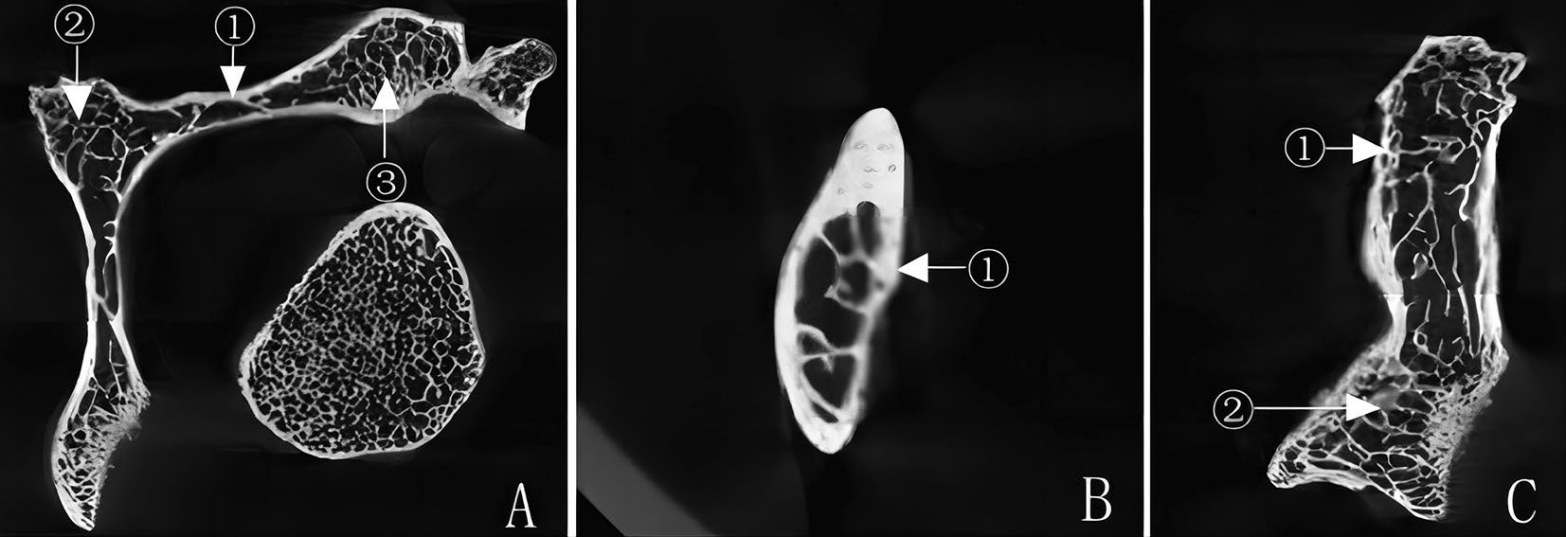
Trabecular arrangement characteristics within the vertebral plate (**A** Cross-sectional: ① vertebral plate ② spinous process ③ articular process. **B** Sagittal plane: ① vertebral plate. **C** Coronal section: ① lamellar trabeculae ② rod-shaped trabeculae).

There are only 1–2 layers of lamellar trabeculae in the vertebral plate near the cortical bone. It then extended to the medullary space and transformed into rod-shaped trabeculae in a radial shape. Rod-shaped trabeculae are connected with oblique or horizontal trabeculae, forming a solid reticular structure. There are more oblique and transverse rod trabeculae parallel to the axis of the lamina (see Fig. 3).

The lamellar trabeculae of the vertebral plate extended into the spinous process near the cortical bone region. It was only 1–2 layers, which then transformed into rod-shaped trabeculae in the medullary space. The trabeculae were discontinuous from the ventral to the dorsal and formed a reticular structure.

**Figure 4:**
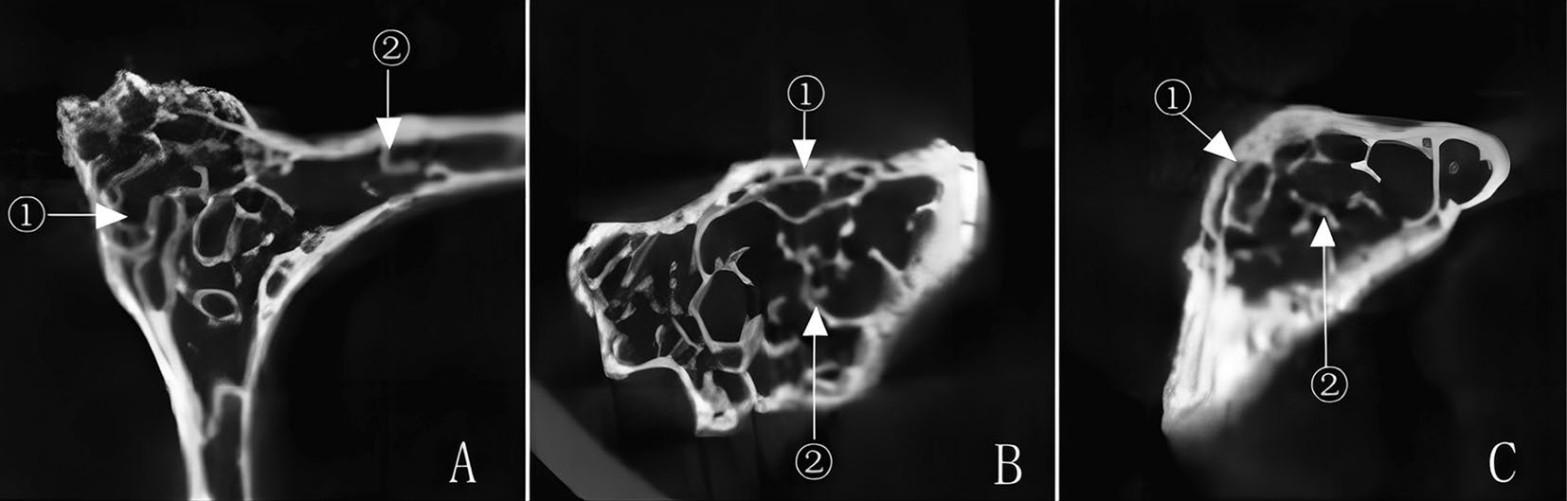
Trabecular arrangement characteristics within the spinous process (**A** Cross section ① spinous process ② vertebral plate. **B** Sagittal plane: ① lamellar trabeculae ② reticular structure; **C** Coronal section ①lamellar trabeculae ② reticular structure).

There were nutrient foramen structures in the pedicle, vertebral plate, and spinous process of the lower cervical spine, which extends from the surface of the cortical bone to the inside of the medullary space (see Fig. 4).

### The changing trend of microstructure

**Figure 5:**
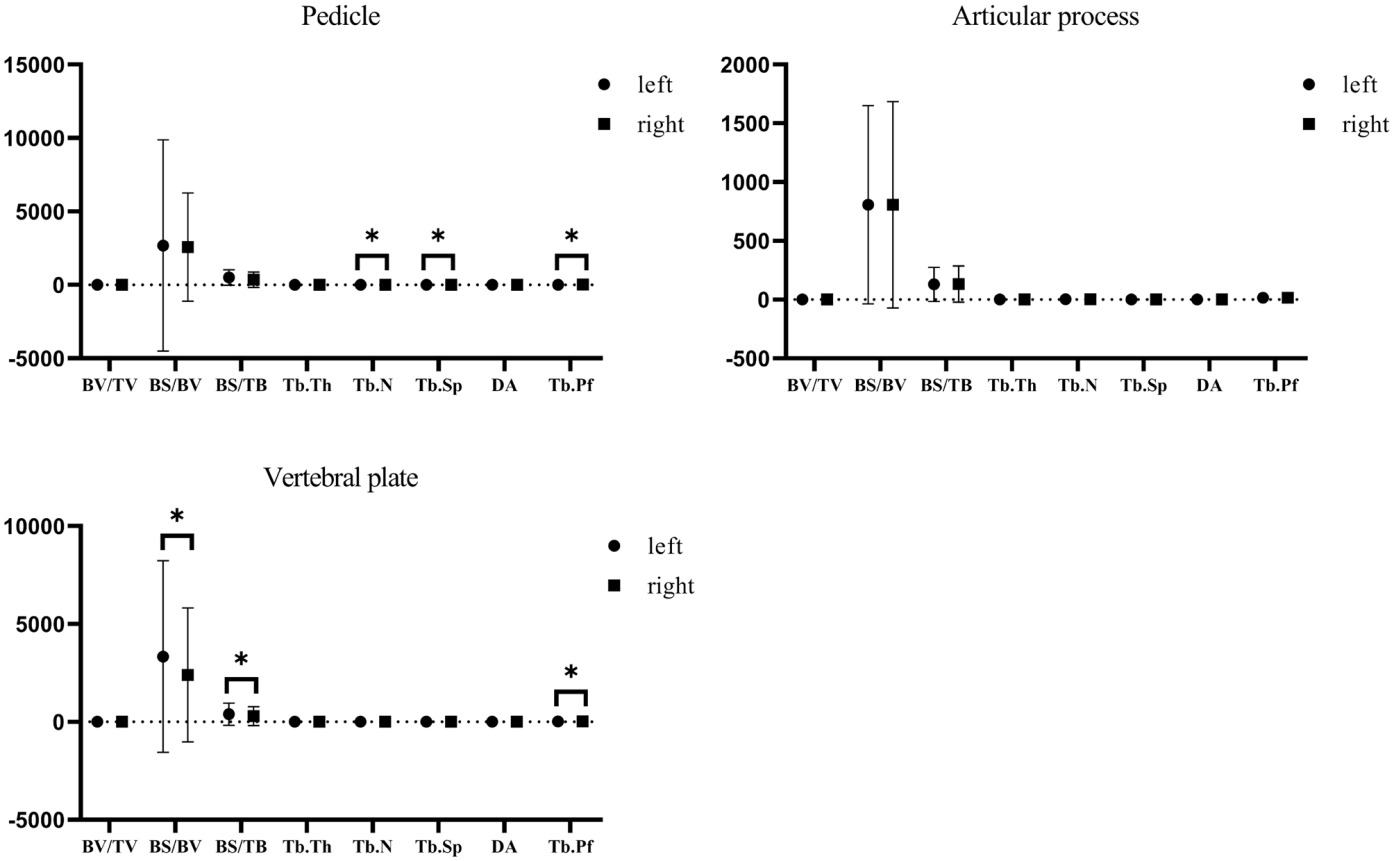
Measurements of C3–C7 pedicle, articular process, and left and right side of the vertebral plate.

We statistically analyzed eight microstructural indicators in the C3-C7 ROIs. The comparison between the left and right sides showed significant differences in the pedicle's Tb. N, Tb. Sp, and Tb. Pf (P < 0.05). The differences in BS/BV, BS/TB, and Tb. Pf in the lamina were also noticeable(P < 0.05), and no significant differences in the rest(P < 0.05) (see Fig. 5).

In ROIs, the minimum BV/TV was in the vertebral plate, and the maximum was in the articular process. There was a significant difference in BV/TV among the pedicle, spinous process, vertebral plate, and articular process (P < 0.05). The trabecular volume fraction was positively correlated with the hardness of the bone, indicating that the articular process bone was relatively dense.

The minimum BS/BV ratio was in the articular process, and the maximum value was in the vertebral plate. Except for no statistical difference between the pedicle and vertebral plate, significant differences were found between the groups (P < 0.05). When the bone surface area was constant, the larger the trabecular volume was, the smaller the trabecular bone volume ratio was. It was consistent with the change law of the microstructural parameters in this experiment.

**Figure 6:**
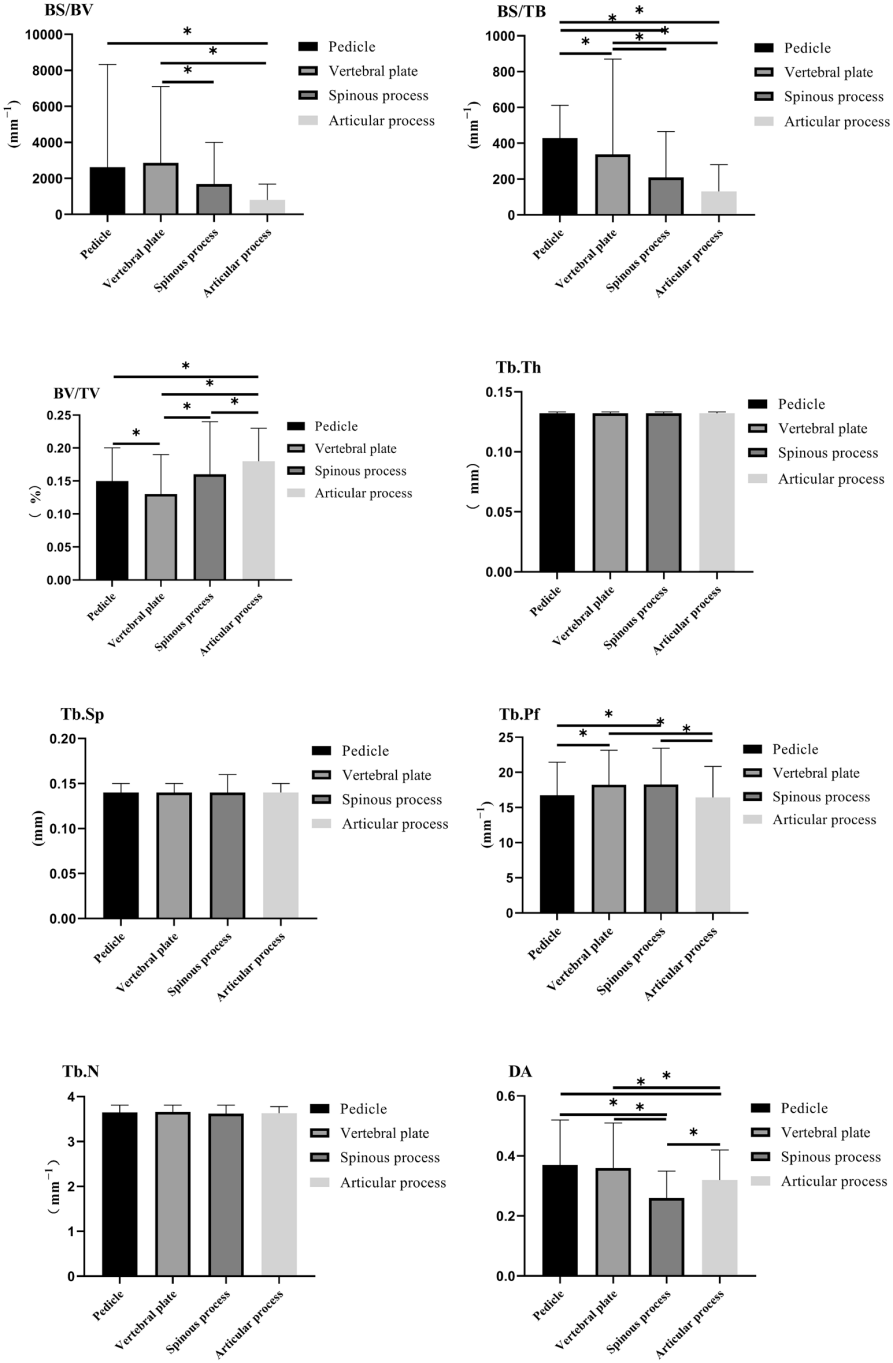
Comparison of measurement results of each index of C3–C7 pedicle, articular process, vertebral plate, and spinous process.

There were significant differences in BS/TB between the articular process, pedicle, and vertebral plate, as between the spinous process and pedicle (P < 0.05). Except for no statistical difference between the pedicle and vertebral plate, there were significant differences in the DA between the rest (P < 0.05). In Tb. Pf, there were significant differences between the pedicle, vertebral plate, and spinous processes, similar to the comparison between the articular processes, vertebral plate, and spinous processes (P < 0.05). There was no significant difference in Tb. Th, Tb. N, and Tb. Sp among pedicle, vertebral plate, spinous process, and articular process (P < 0.05) (see Table 2 and Fig. 6).

**Table 2:** Measurements of each index of C3-C7 pedicle, articular process, vertebral plate, and spinous process.

Index	Pedicle (n = 310)	Vertebral plate (n = 310)	Spinous process (n = 155)	Articular process (n = 310)	F	P
BV/TV	0.15 ± 0.05	0.13 ± 0.06^a^	0.16 ± 0.08^b^	0.18 ± 0.05^abc^	39.42	< 0.0001
BS/BV	2625.77 ± 5704.03	2864.89 ± 4238.74	1690.27 ± 2312.03^b^	807.15 ± 879.78^ab^	17.45	< 0.0001
BS/TB	429.87 ± 182.02	338.10 ± 532.12^a^	209.49 ± 255.57^ab^	131.20 ± 149.24^ab^	49.00	< 0.0001
Tb.Th	0.13 ± 0.001	0.13 ± 0.001	0.13 ± 0.001	0.13 ± 0.001	1.00	0.39
Tb.N	3.65 ± 0.16	3.66 ± 0.15	3.62 ± 0.19	3.63 ± 0.15	3.15	0.02
Tb.Sp	0.14 ± 0.01	0.14 ± 0.01	0.14 ± 0.02	0.14 ± 0.01	1.95	1.00
DA	0.37 ± 0.15	0.36 ± 0.15	0.26 ± 0.09^ab^	0.32 ± 0.10^abc^	29.83	< 0.0001
Tb.Pf	16.75 ± 4.70	18.23 ± 4.93^a^	18.29 ± 5.16^a^	16.44 ± 4.40^bc^	10.97	< 0.0001

^a^vs pedicle, ^b^vs vertebral plate, ^c^vs articular process *P* < 0.05.

## Discussion

The lower cervical spine is prone to loss of spinal stability and spinal nerve injuries in various conditions, such as trauma, deformity, and tumor. The treatment is often with transoperative internal fixation to restore its stability. Commonly used procedures include screwing via the pedicle, the articular process, or the vertebral plate and spinous process^14–18^. Most studies on internal fixation have focused on its stability from a biomechanical perspective. However, most of them have ignored investigating the anatomical microstructure of the vertebrae themselves. In this study, we used micro-CT to observe the 2D and 3D structures of the cortical bone, trabeculae, and bone marrow cavity at the micrometer level. We did the studies from the perspective of the vertebrae's anatomical structure, investigating the trabecular characteristics and alignment patterns in the lower cervical spine and calculating the bone volume parameters accurately. It will provide a basis for further study of the biomechanical characteristics of the lower cervical spine and surgical treatment in the clinic.

During the development of vertebrae, there are three primary ossification centers. The ossification center, located in the transverse process's root, went upward and downward to ossify, forming the superior and inferior articular processes. It went forward to ossify, forming the pedicle and posterolateral part of the vertebral body. Furthermore, it went backward ossification to form lamina and spinous process. The Micro-CT images clearly showed the distribution characteristics of the trabeculae of the lower cervical vertebrae. The lamellar trabeculae blew the cortical bone of the pedicle were dense and thick. The distribution of rod-shaped trabeculae in the medullary space was sparse. Both trabeculae formed a solid meshy structure in the medullary cavity. The characteristic of the pedicle's trabecular structure is adapted to stress in all directions. As the mechanical core of the cervical vertebra, the pedicle focuses on the longitudinal stress from the head and neck and the tension and rotation of the surrounding ligaments so that it forms a dense lamellar bone trabecula. The stress is uniformly dispersed through the radial rod-shaped trabeculae to adapt to the movement function of the cervical spine. Our study showed that trabecular cavities were formed by the unclosed ossification center between the superior and inferior articular processes. The lamellar trabeculae around the cavities were only 1–2 layers in shape, which was the weak area of the structure in this region. When the energy was gathered and transferred here, it was easy to tear. Therefore, lamellar trabeculae around the cavities were most prone to tear. When energy is transmitted along the torn trabeculae, causing fractures, so it is also an essential mechanism of articular process fracture. In our experiment, we observed that the lamellar trabeculae of the vertebral plate extended into the spinous process near the cortical bone region, with only 1–2 layers. Then it converted into rod-shaped trabeculae in solid meshy structures after extending to the cavum medullare. Moreover, there are more oblique and transverse rod-shaped trabeculae parallel to the axis of the lamina, which is the reason for dispersing the stress on the ventral side to the dorsal side.

In this study, the microstructure and morphological parameters of the pedicle, articular process, vertebral plate, and spinous process of the lower cervical vertebra were observed by Micro-CT to evaluate the sclerotin^19–22^. The micro-CT system can calculate the density of the selected three-dimensional ROIs, and the BV/TV can be calculated based on the three-dimensional reconstruction of voxels. Yamada et al^23^ have used high-resolution 3D micro-CT to study the microstructure of lumbar vertebrae. The BV/TV was closely related to the vertebral strength in their compression destruction experiment. But it had a low correlation with the trabecular thickness. The BV/TV in the ROI could predict the maximum and final stress of the bone. Other studies on spinal micro-CT have shown that ROIs with a lower BV/TV also have lower structural stress, which is the weak area of the vertebral structure^24^.

The microstructural differences of ROIs in the lower cervical spine measured in this study showed that the BV/TV significantly differed among them. The value in the vertebral plate was the lowest and the highest in the articular process. The comparative results show that the vertebral plate is relatively osteoporotic, which is a weak point for stress. At the same time, the sclerotin of the articular process is dense, with better biomechanical properties. The overall change trend of the BS/BV ratio is opposite to that of bone trabecular volume fraction. So when the trabecular surface area is constant, the larger the trabecular bone volume, the smaller the BS/BV ratio. It is consistent with the change law of the microstructure parameters of the experiment. Tb. Pf is the parameter of trabecular connectivity. The smaller the value is, the better the trabecular connectivity is. In this study, the articular process and pedicle values are both small, and the articular process is the smallest, indicating that it has better load capacity and osteoporosis is not easy to occur.

## Conclusion

This study used Micro-CT to study the lower cervical pedicle, articular process, vertebral plate, and spinous process. Through the analysis of trabecular parameters, we confirmed that the articular process bone was denser than other parts, and the load capacity was the strongest, followed by the pedicle. Clinical transarticular process or transpedicular screw fixation has a strong holding force. By observing the morphological characteristics of the three-dimensional trabeculae, we found that the unclosed growth center of bone between the superior and inferior articular process is a weak region. Because of the short length of the nail path when placing screws via the articular process, there is poorer resistance to extraction and lateral bending during transarticular internal fixation. The experimental results show that the vertebral plate is relatively osteoporotic, so the use of pedicle screws requires the placement of the nail tract into the spinous process to increase its holding power and resistance to extraction. The results of this trial validate the scientific validity of the currently used clinical methods of internal fixation. Transforaminal screw placement has become a common surgical approach due to its significant superiority over other internal fixation techniques in terms of resistance to extraction force, resistance to lateral bending, and good holding power^10,12,13,16,17^. This study provides strong support for exploring the mechanical characteristics of the lower cervical spine's microstructure and provides a theoretical basis for clinical treatment strategies.

## Data Availability

The datasets used and/or analysed during the current study available from the corresponding author on reasonable request.
